# Circulating non-canonical small non-coding RNAs as novel diagnostic biomarkers for tuberculosis

**DOI:** 10.1128/spectrum.02168-25

**Published:** 2026-02-18

**Authors:** Ping Yang, Shoubin Zhan, Yi Zhu, Zhenbiao Yu, Xiaoshan Zhang, Ning Dong, Shiting Xie, Renjie Miao, Han Shen, Xuemei Wang, Chenyu Zhang, Hong Yan

**Affiliations:** 1 State Key Laboratory of Pharmaceutical Biotechnology, Jiangsu Engineering Research Center for MicroRNA Biology and Biotechnology, NJU Advanced Institute of Life Sciences (NAILS), Nanjing University 12581, Nanjing, China; 2 Department of Laboratory Medicine, The Affiliated Drum Tower Hospital of Nanjing University Medical School, Nanjing, China; 3 Afﬁliated Third Hospital of Zhenjiang to Jiangsu University, Zhenjiang, Jiangsu, China; 4 Department of Clinical Laboratory, The Second Affiliated Hospital of Nanjing Medical University 531909, Nanjing, China; ICON plc, London, United Kingdom

**Keywords:** tuberculosis, biomarker, rsRNA, tsRNA, snoRNA

## LETTER

Tuberculosis (TB) remains a global emergency, despite concerted efforts over the past two decades to develop new diagnostics, drugs, and vaccines supported by continuously expanding pipelines (1). With around 10 million cases annually, the need for cutting-edge diagnostics for TB is immensely exacerbated (2). Although sputum-based GeneXpert MTB/RIF has revolutionized TB detection, its sensitivity plummets to 62% in smear-negative cases and fails to distinguish latent infection from active disease (3, 4). Advanced research highlights the potential of blood-based RNA biomarkers for sensitive detection of TB (5–7); however, the diagnostic potential of circulating non‐canonical small non‐coding RNAs (tsRNAs, rsRNAs, and snosRNAs) remains to be determined.

While our laboratory’s initial discovery established circulating miRNAs as stable serum biomarkers (8), the rich landscape of circulating sncRNAs has since expanded to include non-canonical species such as tsRNAs and rsRNAs, which show emerging diagnostic potential in various diseases (9–12). Beyond their diagnostic utility, these molecules are increasingly recognized as functionally active entities. tsRNAs, generated from precursor or mature tRNAs under cellular stress, are implicated in translational regulation, ribosome biogenesis, and innate immune responses (9, 13). rsRNAs, derived from ribosomal RNA, exhibit dynamic expression during cellular differentiation and immune activation and may play roles in fine-tuning gene expression (14). snoRNAs, traditionally known for guiding RNA modifications, are also processed into stable small RNAs with potential regulatory functions in stress and disease contexts (15). However, a definitive role for these non-canonical sncRNAs in TB diagnosis has not been established. Therefore, we implemented a dedicated analytical framework using SPORTS software (16) to achieve an unbiased profiling of the TB-associated sncRNome, which successfully identified a novel diagnostic signature based on rsRNA, tsRNA, and snoRNA species.

## DYSREGULATION OF NON-CANONICAL sncRNAs IN TUBERCULOSIS

**Fig 1:**
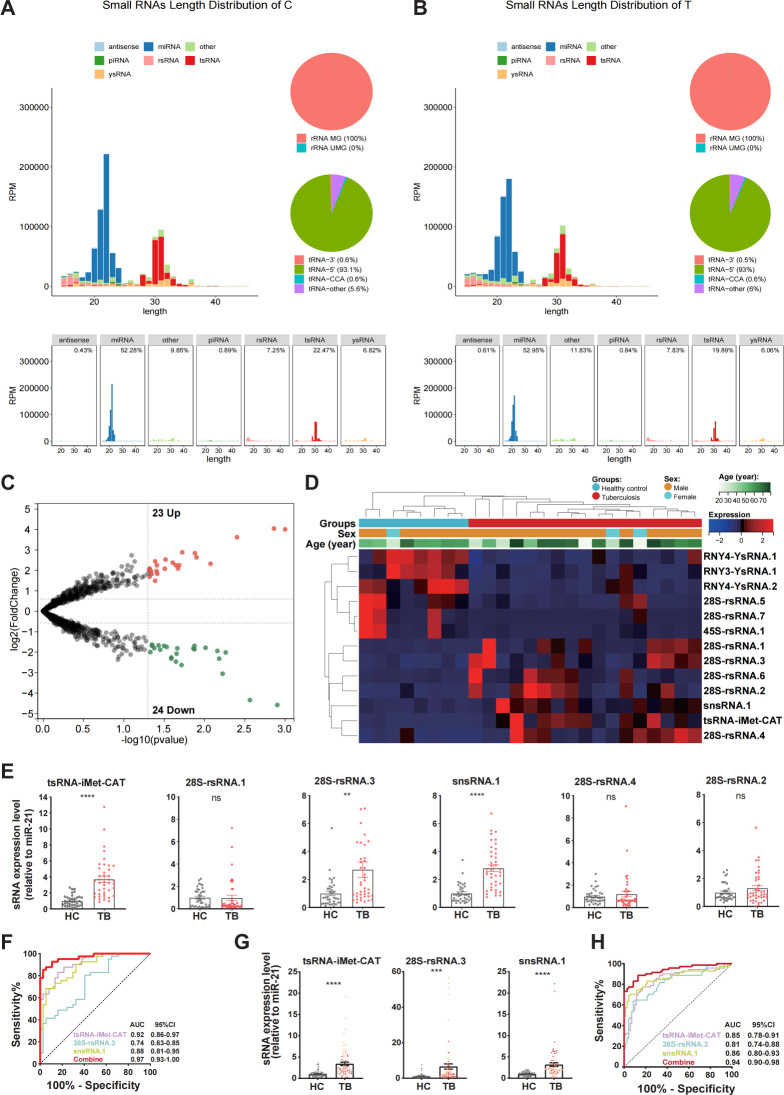
Comprehensive sequencing, validation, and diagnostic evaluation of serum non-canonical small non-coding RNAs in tuberculosis. (**A-B**) Composition and length distribution of serum small RNA species in HC and TB groups. The stacked bar chart depicts the relative abundance of miRNA, tsRNA, rsRNA, piRNA, and snoRNA populations. The line graph represents the read length distribution, highlighting dominant peaks at 21 nt (miRNA) and 30 nt (tsRNA). (**C**) Volcano plot of differentially expressed sncRNAs between TB patients and HCs. The −log₁₀ (*P* value) is plotted against the log₂ (fold change). Red dots denote significantly upregulated sncRNAs (23 species), and blue dots indicate downregulated sncRNAs (24 species), based on filtering criteria (read count >10 in >50% samples). (**D**) Unsupervised hierarchical clustering of significantly dysregulated sncRNAs in TB versus HC groups (|log₂FC| > 2, *P* < 0.02). Rows represent sncRNAs, columns represent individual samples. The color scale reflects Z-score-normalized expression, illustrating a pronounced separation between TB and HC groups and a global increase in sncRNA abundance in TB. (**E**) RT-qPCR validation of six candidate sncRNAs in the training cohort (41 TB vs. 37 HCs). Expression levels were normalized to miR-21 and are presented as mean ± SEM. Significance was determined by unpaired two-tailed Student’s *t*-test; **P < 0.01, ****P < 0.0001, ns stands for not significant (*P* > 0.05). (**F**) Receiver operating characteristic (ROC) curves assessing the diagnostic accuracy of the three-sncRNA signature in the training cohort (41 TB patients *vs* 37 HCs). (**G**) Independent RT-qPCR validation of the three-sncRNA signature in the validation cohort (71 TB vs. 70 HCs). Data are shown as mean ± SEM (***P < 0.001, ****P < 0.0001, unpaired two-tailed *t*-test), confirming robust differential expression in TB. (**H**) ROC analysis of the three-sncRNA signature in the validation cohort (71 TB patients *vs* 70 HCs).

Small RNA sequencing of serum-derived RNA from 18 treatment-naive TB patients and 8 age- and sex-matched healthy controls (HCs) revealed distinct dysregulation patterns in non-canonical small non-coding RNA profiles specific to active *Mycobacterium* TB infection (Fig. 1). Integrated analysis demonstrated canonical small RNA biogenesis signatures, with miRNAs (21 nt) and tsRNAs (30 nt) exhibiting characteristic length distributions that were consistent between patients with TB and HCs, while miRNAs, tsRNAs, and rsRNAs collectively accounted for over 85% of the serum small RNA populations in both cohorts (Fig. 1A and B). Volcano plots were generated from the sequencing data using a stringent filtering criterion that retained only those small RNAs with read counts exceeding 10 in more than 50% of the samples. Based on these criteria, 23 sncRNAs were found to be significantly upregulated, while 24 sncRNAs were downregulated in TB patients compared with HCs (Fig. 1C). Furthermore, heatmap analysis of differentially expressed RNAs between patients with TBs and HCs was conducted using rigorous cutoff criteria (log₂-fold change >2, *P* < 0.02), with hierarchical clustering revealing distinct expression patterns in TB-associated transcriptional signatures. Notably, the heatmap indicated that the overall abundance of sncRNAs was markedly higher in the TB group than in HCs (Fig. 1D).

## DIAGNOSTIC POTENTIAL OF THE sncRNA SIGNATURE IN TUBERCULOSIS

Following rigorous feasibility screening of primer design, six TB‐associated non‐coding small RNAs that exhibited significant upregulation in the TB cohort were selected for preliminary technical validation using quantitative reverse-transcription PCR (qRT‐PCR) in an independent training cohort (41 TB patients; 37 HCs) recruited from Nanjing Drum Tower Hospital. Our analysis identified three small non‐coding RNAs—tsRNA‐iMet‐CAT, 28S‐rsRNA.3, and snsRNA.1—that demonstrated statistically significant differential expression between TB patients and HCs, whereas 28S‐rsRNA.1, 28S‐rsRNA.4, and 28S‐rsRNA.2 showed no significant intergroup variation (Fig. 1E). Receiver operating characteristic (ROC) curve analysis revealed that tsRNA‐iMet‐CAT, 28S‐rsRNA.3, and snsRNA.1 possessed notable discriminative power, with individual area under the curve (AUC) values of 0.92 (95% CI: 0.86–0.97), 0.74 (95% CI: 0.63–0.85), and 0.88 (95% CI: 0.81–0.95), respectively (Fig. 1F). Moreover, their combined signature achieved an AUC of 0.97 (95% CI: 0.93–0.99). In an additional independent large‐scale study (71 TB patients versus 70 HCs), these three small non‐coding RNAs continued to exhibit significant differential expression between TB patients and HCs (Fig. 1G), while maintaining robust diagnostic performance. Specifically, the ROC‐AUC values for tsRNA‐iMet‐CAT, 28S‐rsRNA.3, and snsRNA.1 were 0.85 (95% CI: 0.80–0.89), 0.81 (95% CI: 0.76–0.86), and 0.86 (95% CI: 0.82–0.91), respectively, with the combined signature demonstrating enhanced diagnostic efficacy (AUC of 0.94, 95% CI: 0.90–0.98; Fig. 1H).

## SPECIFICITY OF THE sncRNA SIGNATURE IN DISTINGUISHING ACTIVE TB FROM LATENT INFECTION AND OTHER PULMONARY DISEASES

To evaluate the specificity of the three-sncRNA signature in differentiating active TB from latent infection and other respiratory conditions, we prospectively recruited an independent cohort comprising 48 individuals with latent TB infection (LTBI) and 27 patients with lung cancer. Quantitative PCR analysis revealed distinct expression patterns: snsRNA.1 was significantly elevated exclusively in the active TB group, highlighting its high disease specificity, whereas tsRNA-iMet-CAT and 28S-rsRNA.3 were also upregulated in LTBI and lung cancer patients, suggesting their association with broader inflammatory responses or pulmonary injury (Fig. S1A through C). We further evaluated the diagnostic performance of the combined signature. In discriminating active TB from LTBI, the panel achieved an area under the curve (AUC) of 0.95 (95% CI: 0.91–0.99; Fig. S1D). When differentiating active TB from healthy controls, the AUC was 0.94 (95% CI: 0.90–0.98; Fig. S1E). These results indicate that although individual sncRNAs exhibit varying specificity profiles, the integrated three-sncRNA signature maintains robust diagnostic accuracy, providing a clinically valuable tool for distinguishing active TB from both latent infection and healthy states.

## FUNCTIONAL ENRICHMENT ANALYSIS OF THE THREE-sncRNA SIGNATURE

To obtain initial insights into the potential biological roles of the three-sncRNA signature (tsRNA-iMet-CAT, 28S-rsRNA.3, and snoRNA.1) in TB pathogenesis, we performed bioinformatic functional enrichment analysis on their predicted target genes. Using established target-prediction algorithms followed by Gene Ontology (GO) enrichment, we identified several significantly over-represented molecular functions (Fig. S1 and Fig. S2). The signature was markedly enriched for terms associated with protein post-translational modification (e.g., histone-modifying activity, protein serine/threonine kinase activity), cytoskeletal motor activity, and extracellular matrix organization (e.g., metallopeptidase activity, extracellular matrix structural constituent). These biological processes are fundamentally involved in immune signaling, cell motility, and tissue remodeling—key components of the host response to *Mycobacterium tuberculosis*. Collectively, the enrichment pattern supports the notion that the three sncRNAs may cooperatively regulate a host-response network activated during TB infection, providing a biologically plausible explanation for their strong diagnostic performance and suggesting potential mechanistic roles in TB immunopathology.

## CONCLUSION

In summary, our findings provide compelling evidence supporting the potential of circulating non-canonical small non-coding RNAs as novel diagnostic biomarkers for TB. Despite the highly encouraging nature of our results, the relatively small sample size of the initial discovery cohort (18 TB patients and 8 HCs) should be acknowledged as a limitation, as it may not fully capture the underlying population heterogeneity and could potentially result in missed TB-associated sncRNA markers. While our findings were subsequently validated in two larger independent cohorts, future studies employing larger, multi-center discovery cohorts are still warranted to further confirm the robustness of this three-sncRNA signature and to determine whether additional sncRNA species could further enhance its diagnostic performance. Ultimately, the integration of these biomarkers into diagnostic protocols could enable more precise and timely management of TB, thereby improving patient outcomes and advancing public health.

## Data Availability

The small RNA sequencing data generated in this study have been deposited in the NCBI database and are accessible through accession number PRJNA1259169.

## SUPPLEMENTAL MATERIAL

Supplemental figures spectrum.02168-25-s0001.docx Figures S1 to S3.

ASM does not own the copyrights to Supplemental Material that may be linked to, or accessed through, an article. The authors have granted ASM a non-exclusive, world-wide license to publish the Supplemental Material files. Please contact the corresponding author directly for reuse.
