# Supplementary material for: Circulating non-canonical small non-coding RNAs as novel diagnostic biomarkers for tuberculosis
Source: Microbiol Spectr. 2026 Feb 18;14(4):e02168-25. doi: 10.1128/spectrum.02168-25 (PMC13055209; doi:10.1128/spectrum.02168-25)
Supplement: Supplemental figures — Figures S1 to S3. [file spectrum.02168-25-s0001.docx]

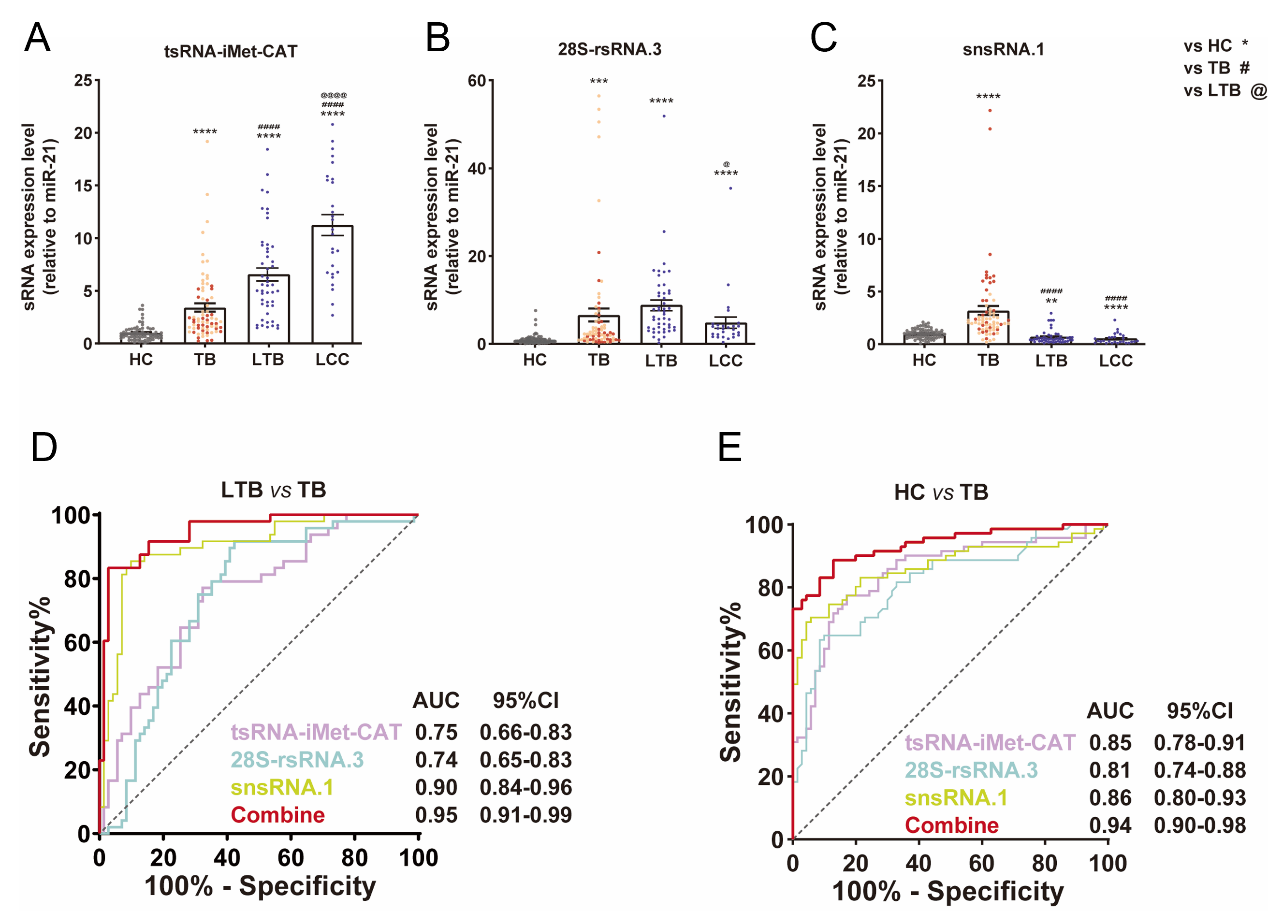


**Supplementary Figure 1. Serum sncRNA expression profiles and diagnostic performance for tuberculosis detection.**
(A) Expression levels of tsRNA-iMet-CAT in healthy controls (HC), individuals with latent tuberculosis infection (LTBI), and patients with active tuberculosis (TB). Expression levels were normalized to miR-21 and are presented as mean ± SEM. ****P* < 0.001.
(B) Expression levels of 28S-rsRNA.3 in HC, LTBI, and TB groups, normalized to miR-21 and presented as mean ± SEM.
(C) Expression levels of snsRNA.1 in HC, LTBI, and TB groups, normalized to miR-21 and presented as mean ± SEM.
(D) Receiver operating characteristic (ROC) curve analysis evaluating the diagnostic performance of the three-sncRNA signature (tsRNA-iMet-CAT, 28S-rsRNA.3, and snsRNA.1) for discriminating active TB from LTBI.
(E) Receiver operating characteristic (ROC) curve analysis evaluating the diagnostic performance of the three-sncRNA signature for discriminating active TB from HCs.


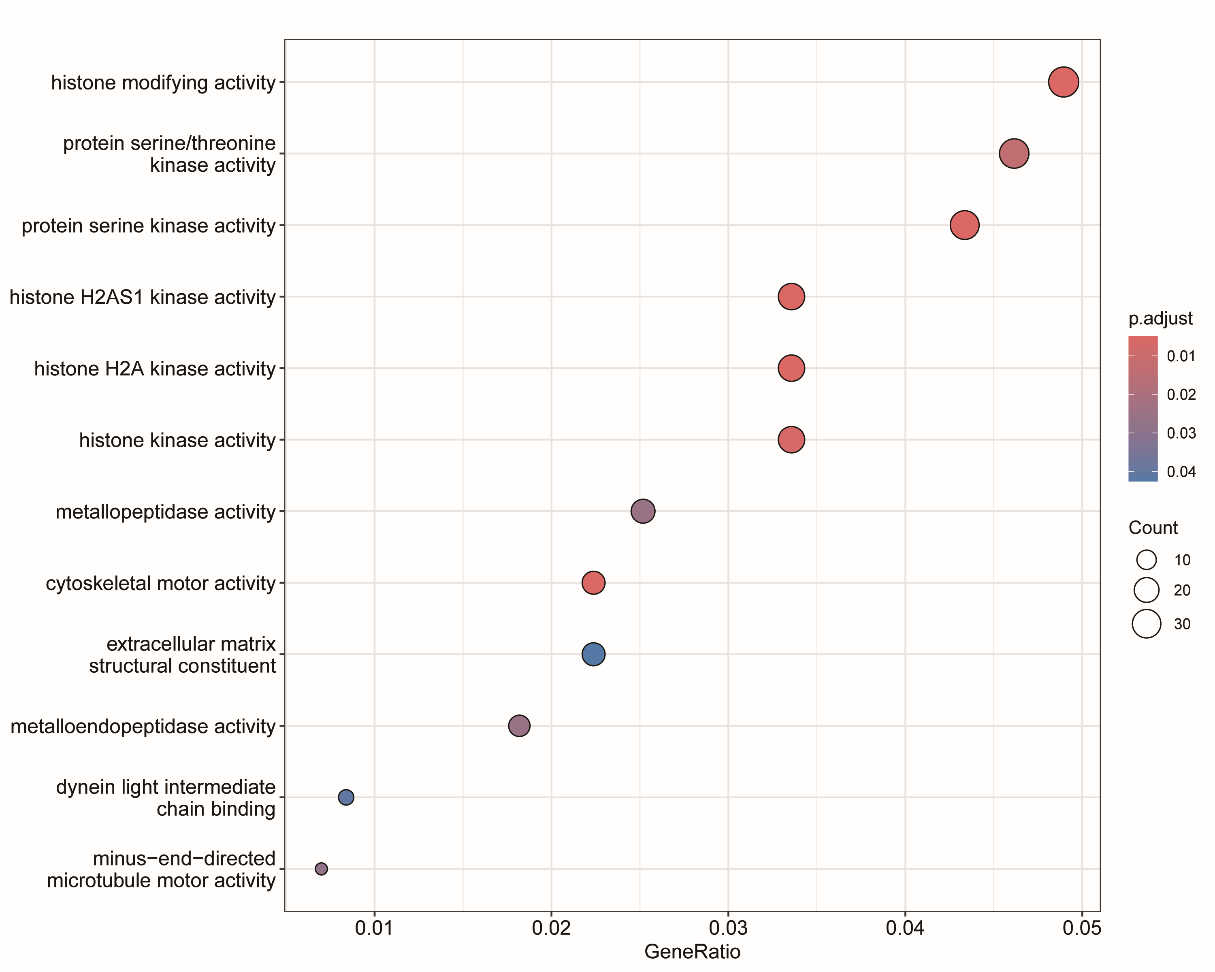


**Supplementary Figure 2. Gene Ontology (GO) enrichment analysis of predicted target genes of the three-sncRNA signature.** Significantly enriched GO molecular function terms (FDR < 0.05) for the predicted targets of tsRNA‑iMet‑CAT, 28S‑rsRNA.3, and snoRNA.1 are shown. Dot size indicates the number of target genes (Count) associated with each term; color gradient represents the adjusted *P* value (*P*.adjust). The x‑axis (GeneRatio) denotes the proportion of target genes mapped to each term relative to the background gene set. Enriched terms are broadly categorized into functions related to protein modification, cytoskeletal activity, and extracellular matrix organization, suggesting a coordinated role in host responses to TB infection.


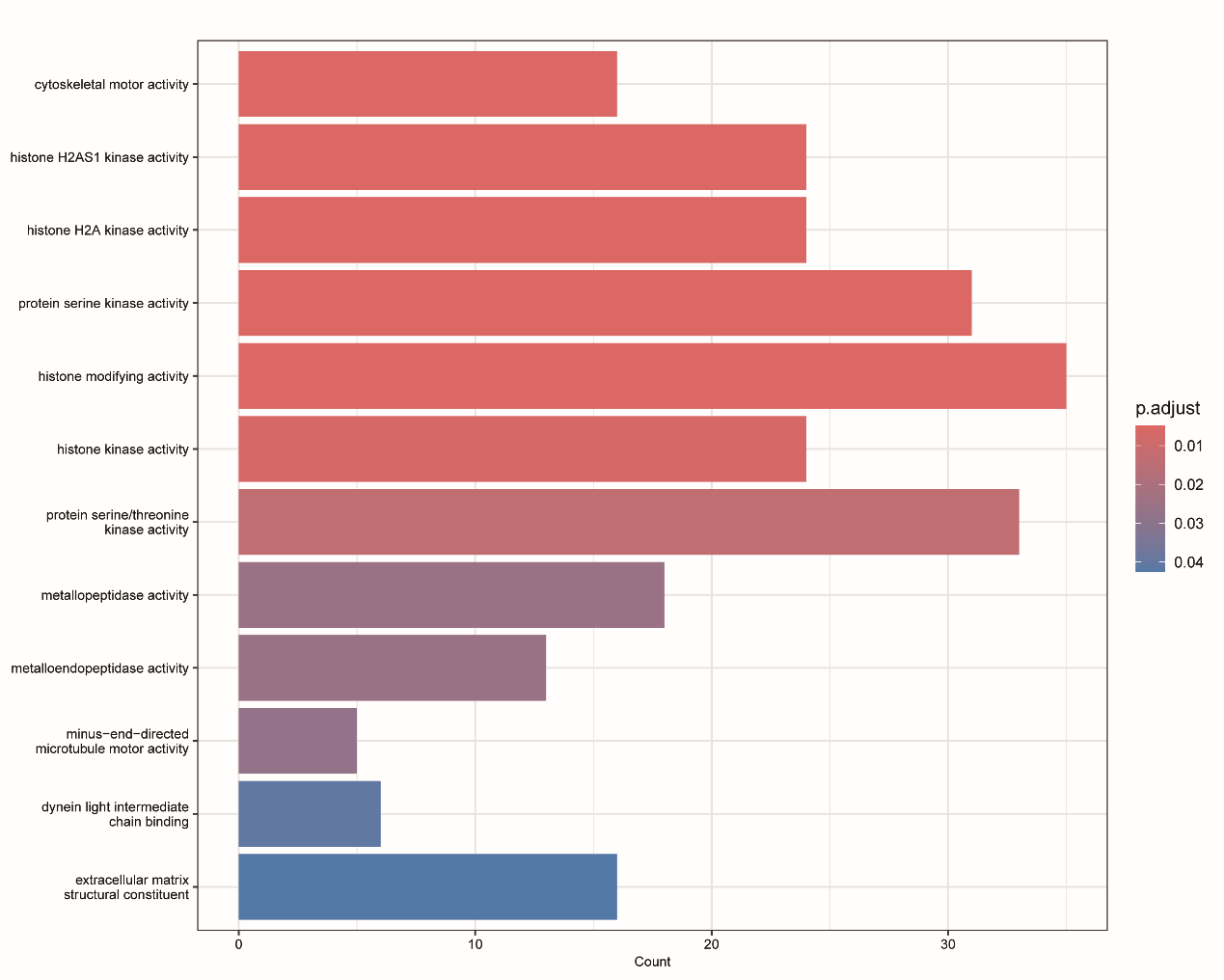


**Supplementary Figure 3. Gene Ontology (GO) enrichment analysis of predicted target genes of the three-sncRNA signature.**
The same set of significantly enriched GO molecular function terms as in Supplementary Figure X is displayed in descending order of gene count. Bar length corresponds to the number of target genes (Count); color indicates the adjusted *P* value (*P*.adjust). This visualization highlights the most abundant functional categories, including cytoskeletal motor activity, histone/protein kinase activity, and metallopeptidase activity, further supporting the involvement of the three-sncRNA signature in immune‑relevant cellular processes during tuberculosis.
